# A randomized clinical trial for comparing the efficacy of desensitizing toothpastes on the relief of dentin hypersensitivity

**DOI:** 10.1038/s41598-023-31616-6

**Published:** 2023-03-31

**Authors:** Ji-Hyun Jang, Soram Oh, Hyun-Jung Kim, Duck-Su Kim

**Affiliations:** Department of Conservative Dentistry, School of Dentistry, Kyung Hee University, 26 Kyungheedae-ro, Dongdaemun-gu, 02447 Seoul, Republic of Korea

**Keywords:** Dental materials, Dental treatments

## Abstract

The 4-week double-blind clinical trial to manage dentin hypersensitivity (DH) using different desensitizing toothpastes was conducted. 53 participants with DH were enrolled in this trial. The participants were randomized into 3 groups: Group N; no active ingredient-containing toothpaste (Pleasia fluoride-free), Group SC; a toothpaste containing strontium chloride (Sensodyne Original), and Group TP; a toothpaste containing tricalcium phosphate (Vussen S). They were instructed to brush their teeth manually for 3 min, 3 times per day for 4 weeks with the allocated toothpastes, and were assessed at baseline (0), 2, and 4 weeks, respectively. Schiff sensitivity score was recorded to 3 different stimuli (air-blast, cold, and acid) at each assessment. Overall DH was also assessed using a visual analog scale (VAS). The longer participants used the toothpastes, the greater reduction in DH in all groups to the three stimuli. Group TP demonstrated significant reduction of DH compared to group N for air-blast and cold stimuli. Group TP showed significantly lower VAS than group N and SC. Tricalcium phosphate containing toothpaste used in this trial was most useful to reduce DH. It can be one of the treatment options that alleviate DH.

Dentin hypersensitivity (DH), referred to as the “common cold of dentistry,” is defined as short, sharp pain arising from the exposed dentin in response to thermal, evaporative, tactile, osmotic, or chemical stimuli that cannot be ascribed to any other form of dental defect or pathology^1^. A wide prevalence range was observed, from as low as 1.3% to as high as 92.1%, in the studies included in the meta-analysis^2^; the study reported the best estimate of DH prevalence to be 11.5%, and the average from all studies was 33.5%^2^. DH can be quite bothersome because of the discomfort and severe disturbances in daily activities. Clinicians must rule out other factors with symptoms similar to those of DH, including caries, tooth fractures, and defective restorations. Dental professionals should identify the factors susceptible to DH, such as periodontal disease, gingival recession, and erosive or abrasive tooth defects.

The management of DH involves decreasing neural transmission and physically occluding dentinal tubules. Potassium nitrate (KNO_3_) was incorporated into desensitizing agents for DH to reduce nerve excitation. Subsequently, this increases the concentration of potassium ions, which is assumed to decrease the excitability of the nerve against the action of sodium ions^3,4^. The other approach is to occlude the already opened tubules or create coagulates inside the tubules^4^. Various in-office and over-the-counter (OTC) preparations are available for DH management. Toothpaste is the most common OTC product used for the treatment of DH. There are several active ingredients in desensitizing products for occlusion of dentinal tubules, including fluoride, strontium chloride, formaldehyde, oxalate salts, and calcium phosphate.

Strontium ions have been studied as conventional active ingredients for DH relief since the 1960s and are the main active components of numerous desensitizing toothpastes. The effectiveness of the product in reducing DH appears to increase with repeated usages^5^. Strontium has attracted attention in the DH treatment because of its chemical similarity to calcium ions^6^. It can strongly adsorb onto all calcified tissues, including the dentin^7^. Strontium ions may consequently accelerate the calcification of minerals, eventually causing obturation of the dentinal tubules^7^. Calcium phosphate minerals are the main inorganic components of dentin and can obstruct tubule orifices during the physiological process of dentin sclerosis. This method mimics the natural process of tooth remineralization. Tung et al. reported that calcium phosphate minerals are ideal candidates for the obstruction of dentinal tubules, owing to their structural similarity to dental hard tissue^8^. In a previous report, using calcium phosphate precipitation, dentinal tubules were occluded predominantly with apatite minerals, not only on the dentin surface but also deep inside the dentinal tubules, to a depth of 10–15 μm from the dentin surface^9^. Being a chemical derivative of calcium phosphate, tricalcium phosphates have been included in toothpastes for DH alleviation, anti-cariogenic effects, and remineralization of caries-like lesions. Therefore, we aimed to investigate the efficacy of toothpastes containing different active ingredients in alleviating DH.

The objective of this prospective randomized clinical trial was to compare the DH-relieving efficacy of a toothpaste containing 19% (w/w) tricalcium phosphate (TP) with that of a positive control containing 10% (w/w) strontium chloride (SC) hexahydrate and a negative control toothpaste with no active ingredient against various stimuli (air, cold, or acid) using Schiff’s sensitivity score and visual analog scale (VAS).

The null hypotheses tested were.

1. The efficacy for relieving DH to air, cold, and acidic stimuli using Schiff’s sensitivity score does not differ significantly according to the type of toothpaste.
2. The subjective VAS scores for DH the do not differ significantly between participants allocated different types of toothpastes.
3. The duration of toothpastes use does not a significant affect DH relief.

## Methods

The participants were randomly grouped as: (1) Group N; no active ingredient-containing toothpaste as negative control (Pleasia fluoride-free; Amorepacific, Seoul, Korea), (2) Group SC; a commercially available toothpaste containing strontium chloride (Sensodyne Original; GlaxoSmithKline, Brendtford, UK), (3) Group TP; a commercially available toothpaste containing tricalcium phosphate (Vussen S; Osstem, Seoul, Korea). The composition of the experimental toothpastes used in this study is listed in Table 1. The participants received their respective desensitizing toothpastes sealed in identical packages without a product label. The participants and examiners were blinded to the names of the allocated toothpaste. One of authors (SO) matched and concealed the alphabetical code (A, B and C) indexed in toothpastes with identical packages to each toothpaste in experimental groups, had the randomized participants list until the end of this trial and supplied the allocated toothpastes to each participant. The participants were instructed to brush their teeth manually for 3 min, 3 times a day, for 4 weeks, and to refrain from using other oral hygiene products and procedures throughout the duration of the study.

**Table 1:** Compositions of experimental materials.

Group	Product (manufacturer)	Composition
**N**	Pleasia fluoride-free (Amorepacific, korea)	Main ingredient: sodium chloride Inactive ingredients: water, glycerin, sorbitol, hydrated silica, hydroxyethyl cellulose, cellulose gum, sodium lauryl sulfate, flavor
**SC**	Sensodyne original (Glaxo SmithKline, korea)	Active ingredient: 10% strontium chloride hexahydrate Inactive ingredients: water, glycerin, sorbitol, calcium carbonate, hydroxyethyl cellulose, silicon dioxide, flavor
**TP**	Vussen S (Osstem, korea)	Active ingredient: 19% Tricalcium phosphate Inactive ingredients: Water, sorbitol, hydrated silica, glycerin, cellulose gum, sodium lauryl sulfate, flavor

### Sample size determination

The sample size was determined according to the previous trials using G Power^10,11^. An effective sample size of 16 subjects in each treatment group would have a power greater than 0.80 (β = 0.2) with an α-level of 0.05, and also have one-sided significance to detect a difference of 1.0 Schiff sensitivity score in air-blast sensitivity score among modalities, assuming a standard deviation of 0.5 Schiff sensitivity score (effective size f = 1.414, number of groups = 3). The allocation ratio was 1. The target sample size comprised of 16 participants in each group, with a total of 48 patients. The sample size was increased to 17 or 18 participants in each group to compensate for the attrition bias.

### Inclusion and exclusion criteria

**Figure 1:**
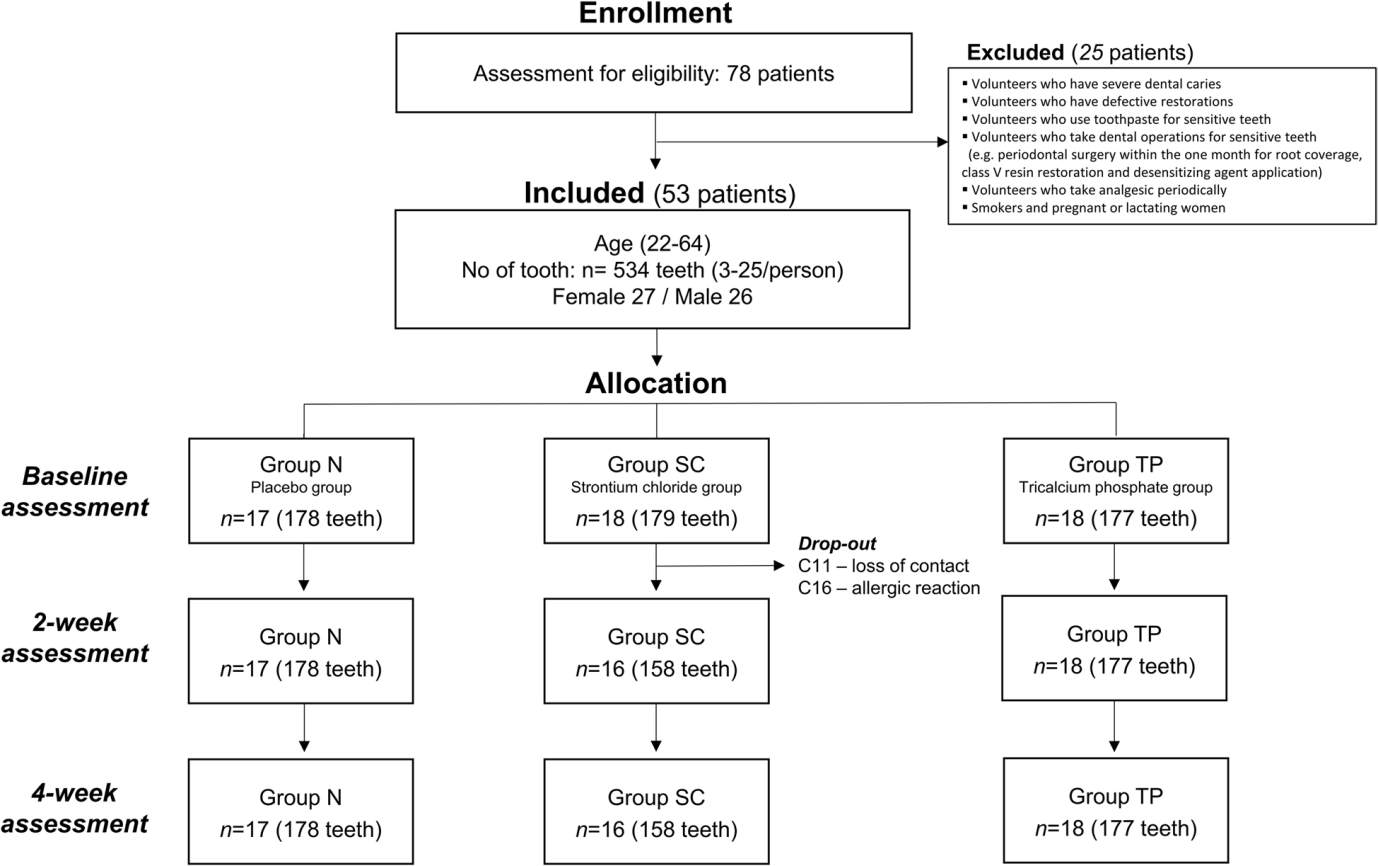
CONSORT flow diagram of this clinical trial.

A total of 112 patients volunteered to participate in this study. Prior to enrollment in the clinical trial, a survey was conducted to identify DH, and participants who did not meet the criteria were excluded. Initially, 78 participants (age, 18–65 y) were enrolled and underwent a primary screening assessment prior to the study using an air-blast test and were evaluated using the Schiff sensitivity score (Fig. 1). Participants who have more than two sensitive teeth with a Schiff sensitivity score of ≥ 2 were included in the study. The criteria for excluding 25 of these participants included severe dental caries, defective restorations, existing use of toothpaste for sensitive teeth, periodic analgesic medication for other illnesses, and dental procedures for DH (e.g., periodontal surgery for root coverage, class V resin filling, and desensitizing agent application) within the past one month prior to the study. Smokers and pregnant or lactating women were excluded from this study. A total of 53 participants were allocated to the three experimental groups. A summary of the demographic characteristics of the participants is presented in Table 2.

**Table 2:** Demographics and schiff scores at baseline.

	Group N	Group SC	Group TP	*p*-value
**Total number of subjects**	17	18	18	-
**Male/Female**	9/8	9/9	8/10	0.877
**Anterior teeth/premolar/molar**	83/62/33	76/56/47	73/58/46	0.421
**Max/Man**	87/91	88/91	90/87	0.923
Mean age (95% C.I.)	32.50 (27.59, 37.41)	31.91 (25.77, 38.05)	32.00 (26.50, 37.50)	0.586
Mean Schiff sensitivity score at screening (95% C.I.)	2.01 (1.65, 2.37)	1.94 (1.53, 2.35)	1.94 (1.53, 2.35)	0.819

CI, confidence interval.

### Randomization

Participants were assigned to each experimental group using a block randomization scheme stratified according to the severity of hypersensitivity. Severity was determined by the average Schiff sensitivity score (mild, 1.167–1.5; moderate: 1.57–1.857, severe: 1.9–2.625) during the screening session. Finally, the 53 participants were randomly divided into three experimental groups. There were 17 subjects in Group N, 18 in Group SC, and 18 in Group TP.

### Clinical assessment

Patients were assessed at baseline and at 2 and 4 weeks. The assessment was conducted by three authors of the study (JJ, HK and DK). At each assessment, the tooth surface was exposed to three different stimuli: air-blast, cold, and application of an acidic solution. Each stimulus was applied according to a standardized protocol, with a gap of 5 min according to Sowinski et al.^12^ Overall DH was assessed using VAS. The examiner was blinded to the experimental group allocation throughout the study.

#### (1) Air-blast

Air was delivered from a standard dental unit air syringe at a distance of approximate 1 cm for 5 s at an operating temperature of 23 °C (±4 °C), directed at the exposed buccal surface of the hypersensitive tooth. The Schiff sensitivity score was used to assess the subject’s response to this stimulus, as follows^13^:

- 0 = Subject did not respond to air stimulus
- 1 = Subject responded to air stimulus but did not request discontinuation of stimulus
- 2 = Subject responded to air stimulus and requested discontinuation or moved away from stimulus
- 3 = Subject responded to air stimulus, considered stimulus to be painful, and requested discontinuation of the stimulus.

#### (2) Cold

Thermal stimuli from the microbrush sprayed immediately with ethyl chloride ice spray (Walter Ritter, Hamburg, Germany) were applied for 1 s to the teeth that had already been tested for air-blast and scored equal to or greater than 1. The Schiff sensitivity score was used to assess the subjects’ response to cold stimuli, following the standard suggested by Schiff et al^13^.

#### (3) Acid

A 20% lemon solution (Fior di Limone; Ital Lemon SPA, Lodi, Italy) was used as the acidic stimulus to assess the DH. The solution was applied for 3 s to the identified tooth surface that had already been tested with air-blast and cold using a microbrush at room temperature and scored using Schiff’s sensitivity score.

#### (4) VAS

At every visit, participants rated subjective DH of the entire dentition based on VAS, with 0, “None” and 10, “Extremely severe” during the usage of their respective toothpastes. VAS scores were recorded in a case record.

### Safety report

Each participant was interviewed regarding the adverse events at each visit. Visual soft-and hard-tissue examinations of the oral cavity were performed at every visit to assess the safety of the products. Spontaneous reports of adverse events were also recorded.

### Statistical analyses

The primary outcomes for this study were the Schiff sensitivity scores for air, cold, and acidic stimuli and VAS scores at baseline (0) and at 2 and 4 weeks. For each test group, the Schiff sensitivity score for each stimulus modality was calculated for each patient and tooth at each assessment. Descriptive statistics were computed for each measurement and the experimental group. The differences in Schiff sensitivity scores between the follow-up and experimental groups were evaluated using a linear mixed-effects model. Statistical significance was set at *P* < 0.05. All computations were performed using the SPSS software (25.0.0, IMB Corp., Armonk, NY, USA).

### Ethics approval

All procedures performed in this study involving human participants were in accordance with the ethical standards of the institutional and/or national research committee and with the 1964 Helsinki declaration and its later amendments or comparable ethical standards. This research was performed and reported according to CONSORT 2010 guidelines. Before the commencement of the study, ethical approval was obtained from the Institutional Review Board (IRB D20-006–001) of Kyung Hee University Dental Hospital.

### Consent to participate and publish

Informed consent was obtained from all individual participants included in the study.

## Results

### Baseline summary

Of the 53 participants enrolled in the study, 2 dropped out due to loss of contact and insistence on allergic reactions; hence, 51 participants completed the study. The study population had a mean age of 32.08 y and a range of 22–65 y. Patient demographics are displayed in Table 2. Demographic features (distribution of men and women, mean age, tooth position, and mean Schiff sensitivity score to air-blast) at baseline were comparable between the control and test groups.

### Efficacy assessment

**Figure 2:**
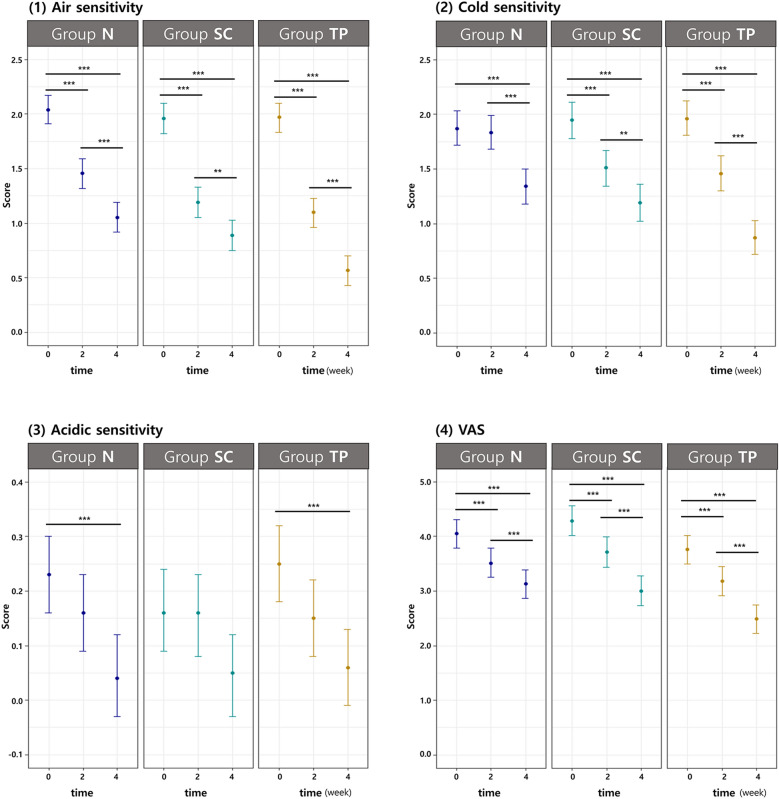
Schiff sensitivity score (least squares means and 95% CI) changes measured at baseline (0), 2-week, and 4-week follow-up to (**1**) air-blast, (**2**) cold, (**3**) acid stimuli and (**4**) general discomfort with visual analog scale. *on top of the bar denote statistically significant differences between groups with* P* < 0.05 level, ** with *P* < 0.01 and *** with *P* < 0.001, respectively. CI, confidence interval.

This study was sufficiently powered (1-β = 0.94) to demonstrate the clinical equivalence of group SC and TP. They were significantly different from group N in the aspect of the reduction of Schiff sensitivity score. A mixed-effects model was used for statistical analysis with adjustments for age, sex, and tooth position (maxilla or mandible; incisor, premolar, and molar). At 2 weeks, all groups exhibited significant desensitization of all stimuli (air-blast; p < 0.0001, cold; p < 0.0001, acid; p = 0.0199) tested in this clinical trial from baseline. At 4 weeks, all groups showed a significant reduction in air-blast, cold, and acidic sensitivity from baseline (p < 0.001) and 2 weeks (p < 0.001). Figure 2 demonstrates the Schiff sensitivity score (least squares means and 95% CI) changes measured at baseline, 2-week, and 4-week follow-up to (1) air-blast, (2) cold, (3) acid stimuli, and (4) VAS.

#### (1) Air-blast

The reduction in air Schiff sensitivity scores of Group TP at 4 weeks from baseline was significantly greater than the reduction in scores of group N (p = 0.0002) (Table 3). There was no significant difference in the reduction in air-blast sensitivity scores between Groups SC and TP (p = 0.2503). Higher desensitization to air-blast stimuli resulted in longer usage of these toothpastes (p < 0.0001).

**Table 3:** Statistics of the Schiff sensitivity score and post-hoc analysis of the mixed effect model for repeated data [mixed effect model; LS mean and 95% CI].

Schiff sensitivity score		Adjusted (LS) mean (95% C.I.)			Bonferroni post hoc (*p*-value)					
		Group N	Group SC	Group TP	Group			Time		
					TP vs N	SC vs N	TP vs SC	0 vs 2	2 vs 4	0 vs 4
Air	Base	2.04 (1.91, 2.17)	1.96 (1.82, 2.10)	1.97 (1.84, 2.10)	**0.0002**	0.0901	0.2503	** < .0001**	** < .0001**	** < .0001**
	2w	1.46 (1.32, 1.59)	1.19 (1.05, 1.33)	1.10 (0.97, 1.23)						
	4w	1.05 (0.92, 1.19)	0.88 (0.74, 1.02)	0.57 (0.44, 0.70)						
Cold	Base	1.87 (1.72, 2.03)	1.96 (1.79, 2.12)	1.96 (1.80, 2.12)	**0.0205**	0.4782	0.6944	** < .0001**	** < .0001**	** < .0001**
	2w	1.83 (1.68, 1.99)	1.51 (1.35, 1.68)	1.45 (1.30, 1.61)						
	4w	1.34 (1.18, 1.50)	1.20 (1.03, 1.36)	0.87 (0.71, 1.03)						
Acid	Base	0.23 (0.16, 0.30)	0.16 (0.09, 0.24)	0.25 (0.18, 0.32)	1.0000	1.0000	1.0000	**0.0199**	** < .0001**	** < .0001**
	2w	0.16 (0.09, 0.23)	0.16 (0.08, 0.23)	0.15 (0.08, 0.22)						
	4w	0.04 (-0.03, 0.12)	0.05 (-0.03, 0.12)	0.06 (-0.01, 0.13)						
VAS	Base	4.05 (3.78, 4.31)	4.29 (4.01, 4.57)	3.75 (3.48, 4.01)	**0.0476**	1.0000	**0.0123**	** < .0001**	** < .0001**	** < .0001**
	2w	3.51 (3.25, 3.77)	3.72 (3.44, 4.00)	3.17 (2.91, 3.44)						
	4w	3.13 (2.86, 3.39)	3.01 (2.73, 3.29)	2.48 (2.21, 2.74)						

LS, least squares; CI, confidence interval; VAS, visual analog scale.

Bold values mean statistical significance.

#### (2) Cold

The Group TP exhibited a significantly higher efficacy of desensitization to cold stimuli than the N group (p = 0.0205). There was no significant difference between Groups TP and SC (p = 0.6944) and Groups SC and N (p = 0.4782).

#### (3) Acid

There were no significant differences among the three groups in terms of the alleviating effects of acidic sensitivity. However, for acidic stimuli, all groups showed a time-dependent decrease in the Schiff sensitivity score from baseline (2 weeks, p = 0.0159; 4 weeks, p < 0.0001).

#### (4) VAS

The mean VAS scores at each follow-up are shown in Table 3. Reduction of hypersensitivity, presented by the participants, was evident during the follow-up period (2 weeks and 4 weeks) (p < 0.0001). Group TP showed a higher reduction in VAS than groups N (p = 0.0476) and SC (p = 0.0123) (Table 3).

### Safety reports

Over the 4-week follow-up period, soft tissue abnormalities which was not presented at baseline were reported by one participant in group SC. After thorough clinical examination and photographic evaluation, the patient consulted a specialist in oral internal medicine. The lesion was localized on the right side of the tongue and appeared to be traumatic; therefore, it was diagnosed as an aphthous lesion, not an allergic lesion. However, the participant voluntarily discontinued the trial.

## Discussion

To relieve DH, various desensitizing modalities have been used at home or professionally applied. Toothpaste is an important agent for the relief of DH. The first desensitizing toothpastes claimed to either occlude dentinal tubules (those that contained strontium salts and fluorides) or destroy vital components within the tubules (those that contained formaldehyde). Currently, most desensitizing toothpastes contain potassium salts such as potassium nitrate, potassium chloride, and potassium citrate. In addition, recent studies reported that a remineralizing toothpaste containing sodium fluoride, bioactive glass (calcium sodium phosphosilicate), and calcium phosphates reduced DH^14–16^.

In this clinical trial, toothpastes containing different active components were evaluated for their DH relief efficacy. As a result, first null hypothesis was rejected. Well-balanced demographics and sufficient statistical power provided evidence of the results among the three groups. Based on this study, Group TP showed no significant difference in the performance of DH alleviation compared with Group SC in the air-blast test (p = 0.250). However, Group TP demonstrated significantly better efficacy of desensitization compared with Group N for air-blast stimuli (p = 0.0002). Furthermore, in response to cold, Group TP exhibited significantly better DH relief than Group N (p = 0.021). In the acidic sensitivity test, all groups showed a significant reduction in DH, especially with longer duration of toothpaste usage (0–2 week, p = 0.020; 0–4 week, p < 0.0001). However, there were no significant differences in the Schiff sensitivity scores among the three groups (p = 1.000). In the VAS score analysis, participants in Group TP reported better DH relief than those assigned to Groups SC (p = 0.012) and N (p = 0.048). Thus, the second hypothesis was also rejected.

In this clinical trial, a greater reduction in DH was observed with a longer duration of toothpaste use in all groups (p < 0.0001). The third hypothesis was rejected. At 2 weeks, all groups exhibited significant desensitization of all stimuli from baseline (air, cold, p < 0.0001; acid, p = 0.020). At 4 weeks, all groups showed a significant reduction in DH in response to all stimuli (air-blast, cold, and acid) from the baseline (p ≤ 0.0001) and 2 weeks (p ≤ 0.0001), respectively.

Strontium chloride was incorporated into the toothpaste assuming that it treated tooth sensitivity by occluding the dentin tubules. It has been used in desensitizing toothpaste because of its compatibility with fluoride. In this study, a positive control product containing 10% (w/w) strontium chloride hexahydrate, marketed as Sensodyne Original, was selected owing to its long-term usage and as a traditional active component since the development of desensitizing toothpaste. However, to date, the data available on its clinical efficacy of DH relief are inconsistent and highly debated^17–19^. Minkoff et al. reported that the therapeutic effects of SC-containing toothpastes were apparent within two weeks and increased continuously for the length of the study by thermal and tactile stimuli in patients with DH^20^. This result was confirmed by the present study. However, fluoro-calcium phosphosilicate bioactive glass containing toothpaste provided a better treatment response than SC-containing toothpaste in relieving DH^21^. Further, a clinical trial demonstrated greater reduction in sensitivity with stannous fluoride-based dentifrice when compared to the SC-based dentifrice in alleviating DH^22^.

The negative control, ‘Pleasia fluoride-free’, did not contain any active ingredients for DH relief. In this trial, considerable reduction was observed in the placebo group. Group N (negative control) showed a reduction of 48.52% for air stimuli and 28.34% for cold stimuli after 4 weeks of usage. Abrasive systems of various particle sizes, including precipitated silica particles and alumina, are present in commercially available toothpastes. The silica abrasive sizes in toothpastes were reported as 3.94–20.4 μm^23^ Thus, dentinal tubule with 1.0–5.0 μm openings might be obstructed by some abrasive particles of toothpaste^24^. The evident placebo effect found in this trial is a response to the intervention itself and consists of complex physiologic and psychological manifestations of the patient’s desire for symptom relief^25,26^. Previous clinical trials on DH treatment reported placebo effects of approximately 30–40%^27,28^. Finally, it is possible that another phenomenon, the Hawthorne effect, may have occurred, contributing to the regression of DH in all groups, including the negative control. The Hawthorne effect refers to the responses to non-interventional procedures that involve frequent recalls during the study period, including improved oral hygiene and patient compliance. Better oral hygiene may have allowed saliva to reach the opened tubules and occlude them with mineral deposition from saliva^29^.

In this clinical trial, Group TP exhibited better DH alleviation than the Group N against air-blast and cold stimuli. In addition, it showed similar effects to Group SC. TP is a biomaterial with a high potential for biological applications^30^. TP has been known to increase salivary calcium levels and is one of the materials that can improve the process of tooth remineralization due to its calcium and phosphate content^31^. It may be speculated that TP induces a response similar to the in vivo remineralization process, thereby contributing to the reduction in DH. A recent meta-analysis revealed that nano-hydroxyapatite toothpastes have a higher efficacy for alleviating DH at two and four weeks compared to calcium sodium phosphosilicate-, potassium-, and strontium–containing toothpastes^32^. In addition, a remineralizing solution with n-HA as the main component induced a significant reduction in enamel surface demineralization. The biomimetic effects of biogenic materials such as n-HA, TP, or CaO might allow the reinforcement of natural hard tissue and aggregation in clusters in the oral environment, which can contribute to reducing DH^33^. HA-based toothpastes induced better enamel remineralization and reduction of DH in white spot lesions compared to conventional fluoride toothpaste in a clinical trial^34^. Moreover, some biogenic material-containing desensitizing toothpastes exhibited anti-inflammatory effect on gingiva^35^.

TP and SC exhibit synergistic DH alleviation with fluoride ions^16,36^. The synergistic action of various forms of fluoride compounds might not be controlled. In this study, we attempted to eliminate the effects of fluoride by prohibiting fluoride–containing desensitizing toothpastes. However, because some regions of the ‘Republic of Korea (South Korea) have enforced a fluorine input project in the water supply,’ and the subject's residence could not be completely controlled, it was difficult to completely rule out the effect of fluoride ions on DH relief. The Decayed-Missing-Filled Teeth index for 12-year-old Korean adolescents improved from 3.30 in 2000 to 1.84 in 2018, which indicates that the oral health status has improved in South Korea^37,38^. Although oral hygiene has improved, prolonged contact between toothbrush bristles and the tooth surface and intensified brushing activity might cause tooth wear, often resulting in DH^39,40^. DH prevalence was previously higher in South Korea than in other countries.

Another limitation of this study is that the concentration of the active ingredient for DH relief was not equal in both toothpastes (Group TP, 19% TP; Group SC, 10% SC) because commercial desensitizing toothpastes were evaluated in this study. Further studies with more controlled variables are required. There may be concerns regarding the four-week duration of our clinical trial. According to Bae et al.^18^, several researchers conducted eight-week clinical trials. To reduce the loss of participants, a period of four weeks was selected, in which the efficacy of sensitive toothpaste was proven^41^. Dentifrices containing 2% strontium chloride and 5% potassium nitrate are efficacious in instantly reducing DH after topical application and after three days of brushing^42^. Clinal studies have shown that n-HA-containing toothpastes are effective for DH relief after two and four weeks^43,44^. However, since the durability or retention of different DH components may vary in the long term, long-term studies are also necessary.

## Conclusion

Within the limitations of this study, it can be concluded that the use of toothpaste containing tricalcium phosphate can reduce DH effectively. If clinicians use it properly, it can be the first option to treatment of initial DH or an alternative to composite resin restoration at cervical lesion.

## Data Availability

The datasets generated and analyzed during the current study are available in patients’ records attending the Conservative Dentistry Department, Kyung Hee University Dental Hospital. The datasets used and/or analyzed during this study are available from the corresponding author on reasonable request and also with the permission of the Conservative Dentistry Department, Kyung Hee University Dental Hospital.

## References

[1] Holland G, Narhi M, Addy M, Gangarosa L, Orchardson R (1997). Guidelines for the design and conduct of clinical trials on dentine hypersensitivity. J. Clin. Periodontol..

[2] Zeola LF, Soares PV, Cunha-Cruz J (2019). Prevalence of dentin hypersensitivity: Systematic review and meta-analysis. J. Dent..

[3] Nagata T (1994). Clinical evaluation of a potassium nitrate dentifrice for the treatment of dentinal hypersensitivity. J. Clin. Periodontol..

[4] Orchardson R, Gillam DG (2006). Managing dentin hypersensitivity. J. Am. Dent. Assoc..

[5] West NX (2007). The dentine hypersensitivity patient-a total management package. Int. Dent. J..

[6] Mathew, R., Hegde, S., Mathew, S., Shruthi, N. & Geevarghese, S. Antimicrobial activity of a remineralizing paste containing Strontium doped Nano hydroxyapatite (Sr-nHAp) with Non Collagenous Protein (NCP) analogue Chitosan–An in vitro study. *Mater. Today: Proc.* (2021).

[7] Addy M, Dowell P (1983). Dentine hypersensitivity-A review: Clinical and in vitro evaluation of treatment agents. J. Clin. Periodontol..

[8] Tung M, Eichmiller F, Gibson H, Ly A, Skrtic D, Schumacher G (1997). Dentin desensitization by in situ formation of calcium phosphate. J. Dent. Res..

[9] Suge T (1995). Effects of fluoride on the calcium phosphate precipitation method for dentinal tubule occlusion. J. Dent. Res..

[10] Sharma D, McGuire JA, Gallob JT, Amini P (2013). Randomised clinical efficacy trial of potassium oxalate mouthrinse in relieving dentinal sensitivity. J. Dent..

[11] Pradeep A, Agarwal E, Naik S, Bajaj P, Kalra N (2012). Comparison of efficacy of three commercially available dentifrices on dentinal hypersensitivity: A randomized clinical trial. Aust. Dent. J..

[12] Sowinski J (2001). Comparative investigations of the desensitising efficacy of a new dentifrice. J. Clin. Periodontol..

[13] Schiff T (1994). Efficacy of a dentifrice containing potassium nitrate, soluble pyrophosphate, PVM/MA copolymer, and sodium fluoride on dentinal hypersensitivity: A twelve-week clinical study. J. Clin. Dent..

[14] Kaufman H, Wolff M, Winston A, Triol C (1999). Clinical evaluation of the effect of a remineralizing toothpaste on dentinal sensitivity. J. Clin. Dent..

[15] Litkowski L, Greenspan DC (2010). A clinical study of the effect of calcium sodium phosphosilicate on dentin hypersensitivity—proof of principle. J. Clin. Dent..

[16] Naoum, S. J., Lenard, A., Martin, F. E. & Ellakwa, A. Enhancing fluoride mediated dentine sensitivity relief through functionalised tricalcium phosphate activity. *Int. Sch. Res. Notices.* **2015** (2015). 10.1155/2015/905019 PMC4897285 27347553

[17] Zappa U (1994). Self-applied treatments in the management of dentine hypersensitivity. Arch. Oral. Biol..

[18] Bae JH, Kim YK, Myung SK (2015). Desensitizing toothpaste versus placebo for dentin hypersensitivity: A systematic review and meta-analysis. J. Clin. Periodontol..

[19] Hu M-L (2018). Effect of desensitizing toothpastes on dentine hypersensitivity: A systematic review and meta-analysis. J. Dent..

[20] Minkoff S, Axelrod S (1987). Efficacy of strontium chloride in dental hypersensitivity. J. Clin. Periodontol..

[21] Aggarwal SD, Borkar A, Borse N, Acharya A (2019). Comparative evaluation of fluoro calcium phosphosilicate, calcium sodium phosphosilicate, and strontium chloride hexahydrate containing dentifrice for the treatment of dentin hypersensitivity: A randomized single-blind study. Int. J. Oral Health Dent..

[22] Vinaya, K. R., Shubhashini, N., Seshan, H. & Kranti, K. A clinical trial comparing a stannous fluoride based dentifrice and a strontium chloride based dentifrice in alleviating dentinal hypersensitivity. *Int. J. Oral Health Dent.* **2** (2010).

[23] de Melo Monteiro GQ (2016). Chromatic and surface alterations in enamel subjected to brushing with desensitizing whitening toothpaste. Eur. J. Dent..

[24] Lenzi, T. L., Camila de Almeida, B. G., Arana-Chavez, V. E. & Raggio, D. P. Tubule density and diameter in coronal dentin from primary and permanent human teeth. *Microsc. Microanal.* **19**, 1445–1449 (2013). 10.1017/S1431927613012725 23947480

[25] Kaptchuk TJ (2009). “Maybe I made up the whole thing”: placebos and patients’ experiences in a randomized controlled trial. Cult. Med. Psychiatry..

[26] Paquette, D. W. & Fiorellini, J. P. Clinical trials and the evaluation of new periodontitis therapies. *Curr. Opin. Periodontol.* 87–98 (1994). 8032470

[27] West N, Addy M, Jackson R, Ridge D (1997). Dentine hypersensitivity and the placebo response: A comparison of the effect of strontium acetate, potassium nitrate and fluoride toothpastes. J. Clin. Periodontol..

[28] Uchida A (1980). Controlled clinical evaluation of a 10% strontium chloride dentifrice in treatment of dentin hypersensitivity following periodontal surgery. J. Periodontol..

[29] Suge T, Kawasaki A, Ishikawa K, Matsuo T, Ebisu S (2006). Effects of plaque control on the patency of dentinal tubules: An in vivo study in beagle dogs. J. Periodontol..

[30] Sanosh K, Chu M-C, Balakrishnan A, Kim T, Cho S-J (2010). Sol–gel synthesis of pure nano sized β-tricalcium phosphate crystalline powders. Curr. Appl. Phys..

[31] Karlinsey RL, Mackey AC, Walker ER, Frederick KE (2010). Surfactant-modified β-TCP: Structure, properties, and in vitro remineralization of subsurface enamel lesions. J. Mater. Sci. Mater. Med..

[32] Hu M-L (2019). Network meta-analysis on the effect of desensitizing toothpastes on dentine hypersensitivity. J. Dent..

[33] Scribante, A. et al. Biomimetic effect of nano-hydroxyapatite in demineralized enamel before orthodontic bonding of brackets and attachments: visual, adhesion strength, and hardness in in vitro tests. *BioMed Res. Int.* **2020** (2020). 10.1155/2020/6747498 PMC7013302 32090106

[34] Butera A (2022). Home oral care with biomimetic hydroxyapatite vs. conventional fluoridated toothpaste for the remineralization and desensitizing of white spot lesions: Randomized clinical trial. Int. J. Environ. Health Res..

[35] Monterubbianesi R (2020). Can desensitizing toothpastes also have an effect on gingival inflammation? A double-blind, three-treatment crossover clinical trial. Int. J. Environ. Health Res..

[36] Li Y (2011). Comparison of clinical efficacy of three toothpastes in reducing dentin hypersensitivity. J. Clin. Dent..

[37] Choi, C. et al. Korea national children’s oral health survey. National Center for Medical Information and Knowledge: Cheongju-si, Korea (2018).

[38] Bernabé E, Sheiham A (2014). Extent of differences in dental caries in permanent teeth between childhood and adulthood in 26 countries. Int. Dent. J..

[39] Bartlett D, Shah P (2006). A critical review of non-carious cervical (wear) lesions and the role of abfraction, erosion, and abrasion. J. Dent. Res..

[40] Splieth CH, Tachou A (2013). Epidemiology of dentin hypersensitivity. Clin. Oral Investig..

[41] Yang, Z.-Y., Wang, F., Lu, K., Li, Y.-H., & Zhou, Z. Arginine-containing desensitizing toothpaste for the treatment of dentin hypersensitivity: a meta-analysis. *Clin. Cosmet. Investig. Dent.* 1–14 (2016). 10.2147/CCIDE.S95660 PMC4708190 26793006

[42] Liu H, Hu D (2012). Efficacy of a commercial dentifrice containing 2% strontium chloride and 5% potassium nitrate for dentin hypersensitivity: A 3-day clinical study in adults in China. Clin. Ther..

[43] Vano M, Derchi G, Barone A, Covani U (2014). Effectiveness of nano-hydroxyapatite toothpaste in reducing dentin hypersensitivity: A double-blind randomized controlled trial. Quintessence Int..

[44] Vano M (2018). Reducing dentine hypersensitivity with nano-hydroxyapatite toothpaste: A double-blind randomized controlled trial. Clin. Oral Investig..

